# Chromosome 2 Interstitial Deletion (del(2)(q14.1q22.1) Syndrome With Novel Skeletal and Central Nervous System Features

**DOI:** 10.7759/cureus.33586

**Published:** 2023-01-10

**Authors:** Rayyan Albarakati, Faroug Ababneh, Mohammed Alharbi, Rakan S Alzahrani, Mohammed F Almutairi

**Affiliations:** 1 Genetics, National Guard Hospital, Riyadh, SAU; 2 Pediatrics, National Guard Hospital, Riyadh, SAU; 3 Genetics and Precision Medicine, King Abdullah Specialized Children’s Hospital, Riyadh, SAU; 4 College of Medicine, King Saud Bin Abdulaziz University for Health Sciences, Riyadh, SAU

**Keywords:** congenital hip dislocation, congenital vertical talus, congenital dislocation of the knee, neural tube defects (ntds), de novo mutation, q2 chromosomal deletion

## Abstract

In this report, we present the case of a Saudi baby boy with a rare de novo interstitial deletion of chromosome 2q14.1-q22.1. His karyotype was confirmed as 46,XY 1(?)q14.2-1q21.3) but further investigation using chromosomal microarray analysis yielded the deletion breakpoints as 2q14.1q22.1. To date, only 10 cases have been reported within or spanning the deletion of the 2q13-2q22.1 region. In comparison to the established cases, our proband shares similar features, such as bitemporal narrowing, deep vein thrombosis, and horseshoe kidney. However, our proband presented with new features which included congenital knee dislocation, congenital vertical talus, bilateral hip dislocation, and myelomeningocele. Moreover, all the reported cases share GLI2 deletion which may reflect the phenotypic features in patients with the deletion of 2q14.1q22.1.

## Introduction

Interstitial deletions of the long arm (q) of chromosome 2 are rare, especially deletions involving the 2q1-q2 region. Greally et al. reported de novo interstitial deletion 2q14.1q22.1 in a four-year-old boy who had a severe delay and post-axial polydactyly [1]. In another study, there was a 1.3 Mb familial deletion at 2q14.2 in which the *GLI2 *gene was the only morbid gene in this region according to Online Mendelian Inheritance in Man (OMIM) [2]. Furthermore, previous studies have reported 10 patients within or spanning deletions of the 2q13-2q22.1 region, and all these patients shared the heterozygous *GLI2 *gene deletion [1-10]. Of note, the omitted region in our proband had 14 OMIM morbid genes. Hence, there is a need to assess whether these implicated genes are responsible for the phenotypic presentation of our proband as well as other similar reported cases.

## Case presentation

The proband was a 19-month-old Saudi boy, a son of healthy consanguineous, first-degree cousins with four other healthy siblings (two girls and two boys). The pregnancy was uncomplicated with unremarkable antenatal ultrasound. He was delivered at term (40+2 weeks) by assisted breech vaginal delivery with a birth weight of 2.62 kg (<fifth percentile), length of 56 cm (<fifth percentile), and occipitofrontal circumference (OFC) of 27.5 cm (<5th percentile). His Apgar score was 4 and 7 at one and five minutes, respectively. He was admitted to the neonatal intensive care unit (NICU) due to respiratory distress and surgical repair for myelomeningocele. During his stay, he was found to have congenital vertical talus and congenital hydrocephalus. He remained in the NICU for 62 days for postoperative observation and ventriculoperitoneal shunt insertion.

**Figure 1:**
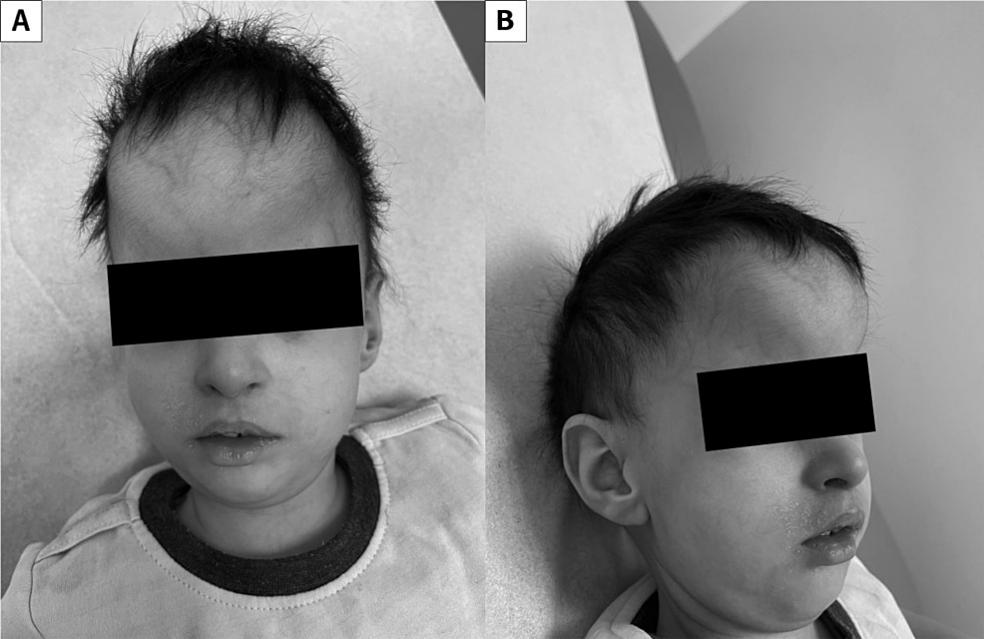
Frontal bossing with bitemporal narrowing, upslanted palpebral fissures (A), and posteriorly rotated ears (B).

**Figure 2:**
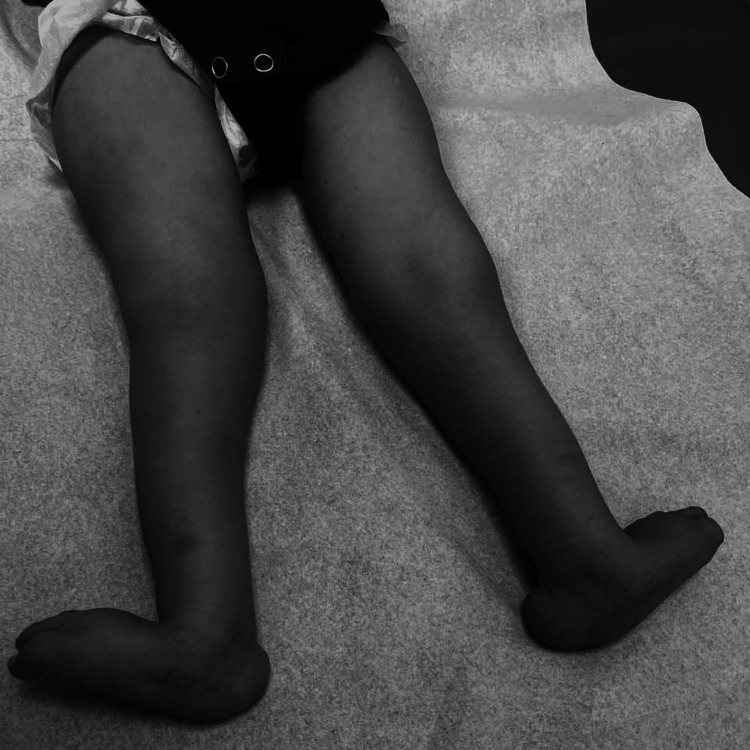
Congenital vertical talus with rocker bottom feet.

On clinical evaluation at 18 months, his weight was 6.5 kg (<fifth percentile), height was 75 cm (<fifth percentile), and OFC was 36 cm (<fifth percentile). On physical examination, he could not hold his head steadily and barely reached for objects. He could coo, smile, and laugh, but could not wave. Further, he presented with frontal bossing with bitemporal narrowing, upslanted palpebral fissures (Figure 1), a small mouth with a high-arched palate, and posteriorly rotated ears (Figure 1). Congenital vertical talus with rocker bottom feet (Figure 2) as well as truncal and peripheral hypotonia along with hyperextensible joints were noted.

Our baseline investigations included a complete blood count, liver and kidney function tests, and serum electrolytes, all of which were normal. Factor V and prothrombin mutations were ruled out. Abdominal ultrasound revealed a horseshoe kidney with left- and right-sided hydronephrosis. Brain MRI showed Arnold-Chiari II malformation, hydrocephalus, and bilateral extradural temporoparietal hematoma. A CT scan was also performed after the treatment of hydrocephalus, which showed a homogeneous hyperdense superior sagittal sinus suggestive of superior sagittal sinus thrombosis, which was confirmed by an MRI venogram.

Karyotyping performed in the neonatal period revealed the interstitial deletion of chromosome 2: 46,XY 1(?)q14.2-1q21.3. Then, chromosomal microarray analysis (CMA) was performed using 250 ng of genomic DNA fragmented, amplified, and hybridized to the array according to the manufacturer’s guidelines. The Cytoscan HD array (Affymetrix) contains 2.7 million markers across the whole genome, including 750,000 single nucleotide polymorphism (SNP) markers, covering 96% of the genes. It enables the detection of copy number variations and/or large deletions/duplications. The results were analyzed using the Chromosome Analysis suite (ChAS, Affymetrix). Copy number variations with a minimum of 25 markers and a size of more than 50 kb (deletions) and 200 kb (duplications) were reported. The SNP component of this array allowed us to analyze the absence of heterozygosity (AOH). The presence of AOH in multiple chromosomes might be consistent with inheritance from a shared ancestor. For homozygous deletions, analysis was performed for all aberrations with at least five aberrant markers and a size of more than 1 kb.

## Discussion

In this report, we present the case of a boy with the heterozygous de novo interstitial deletion of chromosome 2q14.1q22.1. Cytogenetic analysis revealed a 46 XY,del(2) (q1?3q21). Moreover, subsequent CMA analysis pointed out a breakpoint of a 23,497 kb interval between 2q14.1 and q22.1. Although the parents were first-degree cousins, CMA analysis was negative for the same variant. To our knowledge, the interstitial deletion of the long arm of chromosome 2 is rare, with only 10 reported cases within or spanning the deletion of the 2q13-2q22.1 region [1-10]. The proband presented with features that have been reported in these other cases, such as bitemporal narrowing, a horseshoe kidney, and deep vein thrombosis [1-10]. However, our proband presented with a skeletal and neural tube defect that has not thus far been reported with such a deletion. The new features were congenital knee dislocation, congenital vertical talus, bilateral hip dislocation, and myelomeningocele. Therefore, these features expand the phenotype for such a deletion. Interestingly, all the cases reported in the literature with deletion, either within or spanning the 2q13 or 2q22.1 region, share heterozygous *GLI2 *gene deletion, which may indicate that deletion including the *GLI2 *gene accounts for the variability in patients with a de novo interstitial deletion spanning 2q13-2q22.

## Conclusions

We presented the case of a Saudi boy with healthy consanguineous parents with a de novo deletion of 2q14.1q22.1 involving the *GLI2 *gene via CMA. He presented with congenital vertical talus and myelomeningocele that have not been reported in the literature. The patient’s presentation might suggest an extension of the phenotype of this molecular variation. Therefore, the accurate identification of the deletion with its probable wide extensions is necessary to classify this syndrome as a well-known cause for these presentations based solely on the deletion of this region of the chromosome. However, due to the rarity of this mutation, further research is needed to discover the true extent of this deletion with its manifestations.
